# Correction for bias in meta‐analysis of little‐replicated studies

**DOI:** 10.1111/2041-210X.12927

**Published:** 2017-11-21

**Authors:** C. Patrick Doncaster, Rebecca Spake

**Affiliations:** ^1^ Biological Sciences Institute for Life Sciences University of Southampton Southampton UK; ^2^ Geography and Environment University of Southampton Southampton UK

**Keywords:** fixed effect, Hedges’ *d*, Hedges’ *g*, inverse‐variance weighting, ln *R*, random effect, small sample

## Abstract

1. Meta‐analyses conventionally weight study estimates on the inverse of their error variance, in order to maximize precision. Unbiased variability in the estimates of these study‐level error variances increases with the inverse of study‐level replication. Here, we demonstrate how this variability accumulates asymmetrically across studies in precision‐weighted meta‐analysis, to cause undervaluation of the meta‐level effect size or its error variance (the meta‐effect and meta‐variance).
2. Small samples, typical of the ecological literature, induce big sampling errors in variance estimation, which substantially bias precision‐weighted meta‐analysis. Simulations revealed that biases differed little between random‐ and fixed‐effects tests. Meta‐estimation of a one‐sample mean from 20 studies, with sample sizes of 3–20 observations, undervalued the meta‐variance by *c*. 20%. Meta‐analysis of two‐sample designs from 20 studies, with sample sizes of 3–10 observations, undervalued the meta‐variance by 15%–20% for the log response ratio (ln*R*); it undervalued the meta‐effect by *c*. 10% for the standardized mean difference (SMD).
3. For all estimators, biases were eliminated or reduced by a simple adjustment to the weighting on study precision. The study‐specific component of error variance prone to sampling error and not parametrically attributable to study‐specific replication was replaced by its cross‐study mean, on the assumptions of random sampling from the same population variance for all studies, and sufficient studies for averaging. Weighting each study by the inverse of this mean‐adjusted error variance universally improved accuracy in estimation of both the meta‐effect and its significance, regardless of number of studies. For comparison, weighting only on sample size gave the same improvement in accuracy, but could not sensibly estimate significance.
4. For the one‐sample mean and two‐sample ln*R*, adjusted weighting also improved estimation of between‐study variance by DerSimonian‐Laird and REML methods. For random‐effects meta‐analysis of SMD from little‐replicated studies, the most accurate meta‐estimates obtained from adjusted weights following conventionally weighted estimation of between‐study variance.
5. We recommend adoption of weighting by inverse adjusted‐variance for meta‐analyses of well‐ and little‐replicated studies, because it improves accuracy and significance of meta‐estimates, and it can extend the scope of the meta‐analysis to include some studies without variance estimates.

## 1 INTRODUCTION

A meta‐analysis of an effect of interest serves to combine estimates of effect size from across studies, often for the purpose of achieving an overall estimate with more precision than can be obtained from any one study and consequently more power for significance tests (Hedges & Pigott, 2001). Journals of environmental sciences, ecology and evolutionary biology have published an exponentially rising number of meta‐analyses year on year, from 66 in 2001 to 496 in 2015, with a doubling time of 4–5 years (Web of Science search on the topic “meta‐analys*”). For behavioural and ecological studies in particular, meta‐analysis can provide a solution to problems of low replication and pseudoreplication, which afflict costly field studies with little scope for replication within heterogeneous landscapes (Davies & Gray, 2015; Hargrove & Pickering, 1992).

Meta‐analyses usually involve weighting studies to correct for differences in their quality, and weighting is generally considered fundamental to the logic of meta‐analysis (Borenstein, Hedges, Higgins, & Rothstein, 2009; Gurevitch & Hedges, 1999; Koricheva & Gurevitch, 2014). For example, three‐quarters of meta‐analyses in plant ecology are weighted (Koricheva & Gurevitch, 2014). Appropriate weighting prevents less precise estimates from exerting undue and potentially large influence on the evidence accumulated across many studies (Koricheva & Gurevitch, 2013). Weighting still risks overvaluing little‐replicated studies, however, leading to spurious leverage on the cross‐study estimate of effect size and loss of power in random‐effects tests (Hedges & Pigott, 2001; Spake, Ezard, Martin, Newton, & Doncaster, 2015). Field and laboratory research in behavioural ecology is often little replicated (Jennions & Møller, 2003), and meta‐analyses in ecology and evolutionary biology routinely include studies with many‐fold differences in replication (Figure 1).

**Figure 1:**
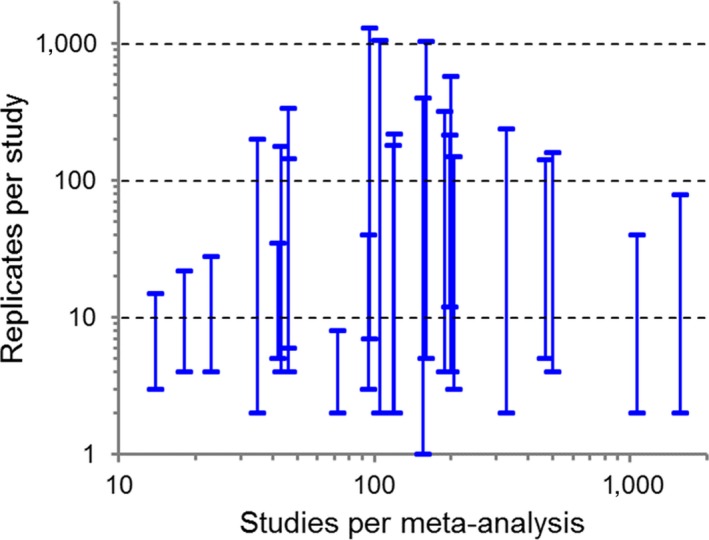
Minimum and maximum replicates per study as a function of the number of studies per meta‐analysis. Data were extracted from a systematic review by Koricheva and Gurevitch (2014) of 322 meta‐analyses published in the field of plant ecology between 1996 and 2013 that provided source datasets. Here, showing the 25 most recent papers to specifically include sample size data

Meta‐analyses conventionally weight each study *i* by the inverse of its observed error variance: $1/{\hat{v}}_{i}$ for fixed‐effects tests, or $1/\left({\hat{v}}_{i}+T^{2}\right)$ for random‐effects tests, where *T* ^2^ estimates the between‐study variance. This weighting aims to minimize the variance in the meta‐estimate of effect size, thereby maximizing its precision (Hedges, 1981). The precision of a meta‐estimate is the closeness of repeated measures of the effect to each other, and therefore a measure of repeatability. Often the ultimate aim of meta‐analysis, however, is accurate meta‐estimation of the effect (Lajeunesse, 2010). Accuracy is the difference between the meta‐estimate of effect size and its true global value, with a small difference signifying high accuracy. In the absence of systematic bias, greater precision leads to higher accuracy.

In this paper, we reveal that bias in any estimator will inevitably creep in to conventionally weighted meta‐analysis, as a consequence of inverting the unbiased study‐level estimates of error variance. Symmetrical variability in the sample ${\hat{v}}_{i}$ estimating a true population *v*_*i*_ increases geometrically with reducing study‐level replication, and accumulates asymmetrically across the weighted studies, to cause underestimation of the meta‐level effect size or error variance (the meta‐effect or meta‐variance). This bias in turn has a systematic influence on the significance of the meta‐effect. Hedges (1982, 1983) recognized a small‐sample bias associated with variability in ${\hat{v}}_{i}$ estimating *v*_*i*_, for precision‐weighted meta‐estimation of the standardized mean difference. Likewise, Hedges, Gurevich, and Curtis (1999) and Lajeunesse (2015) reported small‐sample bias in meta‐estimation of the log response ratio. To our knowledge, however, no study has quantified the replication dependence of the variability in ${\hat{v}}_{i}$ that biases precision‐weighting on all estimators. Here, we enumerate this dependency, with the objective of adjusting the inverse‐variance weighting to account for the deviations of ${\hat{v}}_{i}$ from *v*_*i*_ due specifically to sample size. We aim thereby to improve the accuracy and precision of meta‐analyses, particularly those that include little‐replicated studies. We use simulations of meta‐analyses on known parameter values to compare between conventional and adjusted weightings and a weighting on replication only.

## 2 MOTIVATING EXAMPLES OF THE PROBLEM

The precision and the accuracy of an effect‐size estimate both depend on the error variance *v*. Precision increases with the inverse of *v*, and the probability of losing or gaining accuracy is an inverse circular function of *v* (Gauch, 2006; Hedges, 1981). Consider an effect of interest defined by a population mean μ with variance σ^2^ of random observations drawn from a normal distribution. For *n* replicate observations, it has *v* = σ^2^/*n*. Given a highly replicated study *A* and a little‐replicated study *B* of this population, the larger *v*_*B*_ than *v*_*A*_ raises the magnitude of inaccuracy, $\left|\overset{¯}{Y}-\mu \right|$, in the sample mean $\overset{¯}{Y}$of study *B* above that of study *A* with probability (2/π) arctan $\left(\sqrt{v_{B}/v_{A}}\right)$ (Gauch, 2006; Webb, Smith, & Firag, A, 2010). For example, a study *B* with the same σ^2^ as study *A* but half (or quarter) its replication has a 61% (or 70%) probability of greater inaccuracy due to *v*_*B*_/*v*_*A*_ **= 2 (or 4). In comparison, equal replication has a 50% probability, meaning an equal likelihood of more or less inaccuracy. The relative precision of the study *B* estimate set by *v*_*B*_/*v*_*A*_ thus determines its probability of losing accuracy.

This 1:1 correspondence of precision with accuracy at the study level would apply to a meta‐estimate based on inverse‐variance weighting only if the population variance σ^2^ were estimated precisely by the sample variance *s* ^2^ among observations. This is because the sample variance *s* ^2^ (and not the unknown σ^2^) determines the estimate of the error variance: ${\hat{v}}_{i}=s^{2}/n_{i}$, and hence the study weight of $1/{\hat{v}}_{i}$ for meta‐analysis. The sample variance *s* ^2^, however, is subject to variability due to sampling error in estimating σ^2^, which rises as an inverse function of *n*. This variability is described in Figure 2a, showing it to be equal to twice the sampling error *v* in $\overset{¯}{Y}$ estimating μ**.** A deviation of *s* ^2^ below or above σ^2^ will cause a weighting by $1/{\hat{v}}_{i}$ to over‐ or undervalue the precision, and hence accuracy of the effect‐size estimate for the study. If *s* ^2^ is estimated with unbiased variability around σ^2^, as we may expect, even symmetrically distributed deviations will accumulate asymmetrically in the estimation of the meta‐variance to cause its undervaluation. In order to understand how this meta‐level bias arises from unbiased study‐level $s_{i}^{2}$, we need to understand the relationship of the estimated meta‐variance to the true meta‐variance.

**Figure 2:**
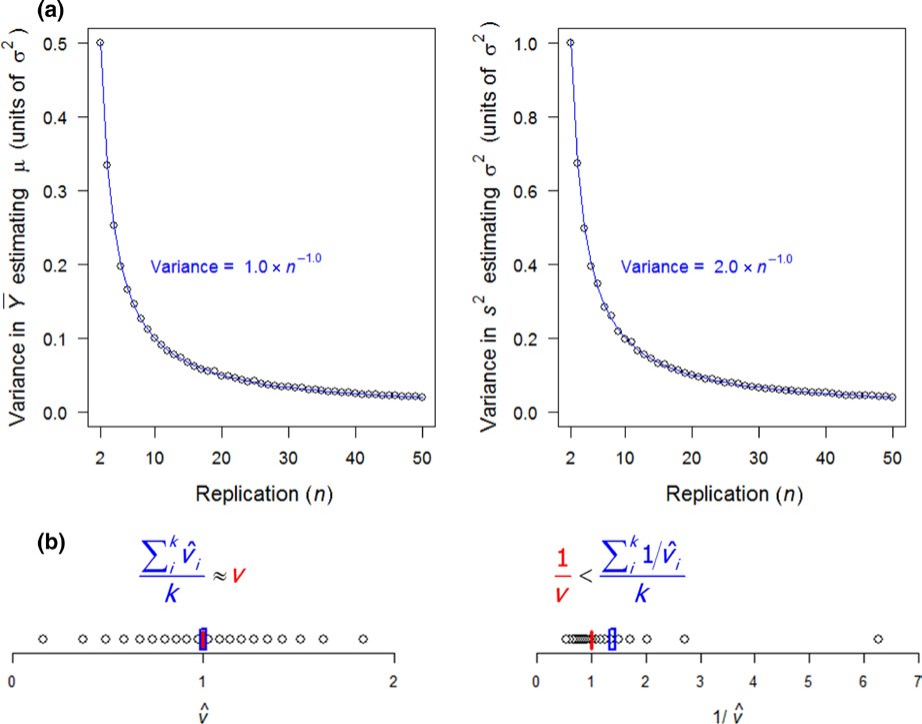
Replication‐dependent variability in estimating the mean and variance. (a) Points show variances in $\overset{¯}{Y}$ (left‐hand graph) and in *s* ^2^ (right‐hand graph) from 10,000 replicate samples of *n* observations of a normal distribution with mean μ and *SD* σ. The error in $\overset{¯}{Y}$ estimating μ is the ‘error variance’: *v* = σ^2^/*n* (left‐hand graph); the error in *s* ^2^ estimating σ^2^ is 2*v* (right‐hand graph). (b) Example of *k* = 20 observations of ${\hat{v}}_{i}$ sampled from a normal distribution around *v* = 1 (left‐hand graph); inversion imposes right skew, with the distribution of $1/{\hat{v}}_{i}$ having mean $(\sum_{i}^{k}1/{\hat{v}}_{i})/k$ exceeding 1*/v*, in this case by 39% (right‐hand graph). Consequently, the true meta‐variance, *v/k*, exceeds the estimated meta‐variance, $1/\sum_{i}^{k}1/{\hat{v}}_{i}$ by the same proportion

The meta‐variance is estimated by the inverse of the sum of weights across the *k* studies included in the meta‐analysis (i.e. the inverse of $1/{\hat{v}}_{1}+1/{\hat{v}}_{2}+\ldots +1/{\hat{v}}_{k}$ for a fixed‐effects meta‐analysis). The true meta‐variance is likewise given by the inverse of 1/*v*_1_ + 1/*v*_2_ + … + 1/*v*_*k*_. Consider the simplest scenario of all studies having the same true *v* = σ^2^/*n*, which yields a true meta‐variance of *v/k*. We now assume that each $s_{i}^{2}$ is an unbiased estimator of σ^2^, such that the arithmetic mean of ${\hat{v}}_{1}+{\hat{v}}_{2}+\ldots +{\hat{v}}_{k}$ will coincide with the true *v* on average. It then follows directly that the estimated meta‐variance must undervalue the true meta‐variance: $1/\sum_{i}^{k}1/{\hat{v}}_{i}<v/k$. This inequality applies to any set of positive numbers ${\hat{v}}_{i}$ that are not all equal, because such sets have a harmonic mean, $k/\sum_{i}^{k}1/{\hat{v}}_{i}$, that is always less than the arithmetic mean, *v* (Xia, Xu, & Qi, 1999). In effect, inversion of the ${\hat{v}}_{i}$ imposes right skew on their distribution, as illustrated in Figure 2b by an example. The magnitude of bias in estimating the meta‐variance rises with the magnitude of variability in ${\hat{v}}_{i}$ around *v*, which itself depends only on *n* for a given σ^2^ (Figure 2a).

We will show for example how 20 studies, each with *n*_*i*_ = 10 for estimating a one‐sample mean, undervalue the meta‐variance by 21% on average, regardless of the magnitude of σ^2^ estimated by the $s_{i}^{2}$. Simulations will further demonstrate that meta‐estimates are likewise undervalued with study‐specific *n*_*i*_ and σ_*i*_^2^ for both one‐ and two‐sample designs, and for fixed‐ and random‐effects tests, as a direct consequence of the *n*‐dependent variability in $s_{i}^{2}$ estimating σ_*i*_^2^. Although some estimators have more elaborate formulations of error variance than σ_*i*_^2^/*n*_*i*_, we will show that they too express similar magnitudes of undervaluation in either the meta‐variance or the meta‐effect.

The bias in meta‐estimation would be eliminated by weighting studies on *n*_*i*_ instead of the conventional $1/{\hat{v}}_{i}$. Indeed, *n*‐weighting is often the best alternative to inverse‐variance weighting when contributing studies provide no estimates of within‐study variance $s_{i}^{2}$ (Brannick, Yang, & Cafri, 2011; Marín‐Martínez & Sánchez‐Meca, 2010). Although *n*‐weighting can provide an unbiased estimator of effect size, it has the considerable disadvantage of enforcing the same value of unity for all studies on $s_{i}^{2}$ and any other components of error variance not attributable to study‐specific replication, which generally rules out sensible estimation of a meta‐variance (Hedges, 1983). When estimates of $s_{i}^{2}$ are available, they provide valuable information on study precision independently of *n*_*i*_. Thus, an inverse‐variance weighting on highly replicated studies can be expected to fulfil its intent in improving precision and accuracy of the meta‐effect and meta‐variance compared to *n*‐weighting by virtue of its greater richness of information. Although we have seen how the same weighting introduces bias when applied to little‐replicated studies, our knowledge of the *n*‐dependent variability in $s_{i}^{2}$ that causes the bias opens up the possibility of addressing it. In the next section, we develop a method of adjusting the conventional inverse‐variance weighting to reduce or eliminate the bias caused by low replication. We then use simulations to evaluate the adjustment against the conventional inverse‐variance weighting and *n*‐weighting.

## 3 MATERIALS AND METHODS

We develop adjusted weightings for commonly used estimates of effect size based on means and normally distributed residuals. We assume that the primary studies (hereafter ‘studies’) for a meta‐analysis have been collated by systematic review and filtering to remove biases due to differences in treatment factors and levels, scales of sampling or response types (Spake & Doncaster, 2017). For example, in a meta‐analysis of experiments testing the effects of a neonicotinoid insecticide on honeybees, we assume that all studies measure the same insecticide and treatment levels (e.g. dose vs. control), allocated to sampling units of bee colonies drawn from the global population of honeybees, with each colony measured for the same type of response among its bees. A random‐effects meta‐analysis may relax these assumptions, if different types of effect (or response) are randomly sampled from a population of types with a normal distribution of effects (or responses). We further assume that studies have a random allocation of treatment levels to sampling units; otherwise pseudoreplication inflates the precision of the study estimate, and its value if the effect size is standardized against study variance (Halme et al., 2010).

### 3.1 Derivation of inverse‐variance weighting and its adjustment

The inverse‐variance weighting has a well‐established methodology (e.g. Borenstein, Hedges, Higgins, & Rothstein, 2010; Borenstein et al., 2009; Gurevitch, Curtis, & Jones, 2001; Hedges & Olkin, 1985), which we adhere to for commonly used one‐sample estimators (such as a mean or mean difference) and two‐sample estimators (such as a response ratio between two treatments). Generally for all estimators, a fixed‐effects meta‐analysis treats all studies as estimating the same effect δ, making the error variance *v*_*i*_ the only source of error in the estimation of δ. A random‐effects meta‐analysis treats each study as having its own δ_*i*_, from a normally distributed population with between‐study variance τ^2^ around a global average δ. The estimation of δ has precision determined by an overall variance equal to *v*_*i*_ + τ^2^, estimated by ${\hat{v}}_{i}+T^{2}$. Table 1 summarizes the terminology. Table 2 details the parametric and estimated study‐level effects and variances for estimators including the log response ratio ln*R*, and Hedges’ *g* for a standardized mean difference (SMD).

**Table 1:** Glossary of parameters and their estimates for meta‐analysis with inverse‐variance weighting. Fixed‐effects and random‐effects meta‐analyses assume τ equal to zero and exceeding zero respectively. Square brackets illustrate meta‐analysis of a one‐sample mean; for other estimators, see Table 2

Measure	Population parameter	Sampled estimate
Within the *i*th study, replicate [*n*_*i*_] normally distributed observations of the response		
Effect size	δ_*i*_[ = μ_*i*_]	${\hat{\delta }}_{i}\left[={\overset{¯}{Y}}_{i}\right]$
Variance in observations^a^	$\sigma_{i}^{2}$	$s_{i}^{2}$
Within‐study error variance^b^	$v_{i}\left[=\sigma_{i}^{2}/n_{i}\right]$	${\hat{v}}_{i}\left[=s_{i}^{2}/n_{i}\right]$
Amongst *k* studies, *k* normally distributed observations of δ_*i*_ around δ		
Variance in observations	τ^2^	*T* ^2^
Between‐study error variance	τ^2^/*k*	*T* ^2^/*k*
Meta‐analysis of the *k* studies		
Study weighting		$W_{i}=1/\left({\hat{v}}_{i}+T^{2}\right)$
Meta‐effect size	δ	$\sum_{i}^{k}W_{i}\cdot {\hat{\delta }}_{i}/\sum_{i}^{k}W_{i}$
Meta‐variance	$1/\sum_{i}^{k}1/\left(v_{i}+\tau^{2}\right)$	$1/\sum_{i}^{k}W_{i}$
Meta‐analysis when all *k* studies have, or are assumed to have, equal precision (invariant σ^2^, *n*)		
Meta‐effect size	δ	$\sum_{i}^{k}{\hat{\delta }}_{i}/k$
Meta‐variance	*v*/*k* + τ^2^/*k*	${\hat{v}}_{\mathrm{pooled}}/k+T^{2}/k$

a Also known as the ‘population variance’.

b Also known as the ‘sampling variance’.

**Table 2:** Alternative estimators of study‐level effect sizes and their variances. The mean is the one‐sample mean or mean difference. Ln*R* is the two‐sample log response ratio for means μ_1_, μ_2_ ≫ 0 (Hedges et al., 1999; Lajeunesse, 2015). SMD is the two‐sample standardized mean difference estimated by Hedges’ *g* = *J·d*, with $d=({\overset{¯}{Y}}_{1}-{\overset{¯}{Y}}_{2})/s$, small‐sample correction: $J=\Gamma \left(q/2\right)/[\sqrt{q/2}\cdot \Gamma \left(\left(q-1\right)/2\right)]\approx 1-3/\left(4q-1\right)$, and $\tilde{n}=n_{1}\cdot n_{2}/\left(n_{1}+n_{2}\right)$ (Hedges, 1981). The estimate $\hat{v}$ for SMD has a denominator of the second term given by 2(*n*_1_ + *n*_2_), which assumes that *n*'s become large with δ fixed; an alternative denominator given by 2(*n*_1_ + *n*_2_ − 2) assumes that *n*'s become large with $\sqrt{n\delta }$ fixed (Borenstein et al., 2009; Hedges, 1981), which we used in simulations for the mean‐adjusted $\hat{v}$ when |δ| ≤ 1

Estimator	Effect size		Error variance			d.f.*q*
	Population, δ	Estimate, $\hat{\delta }$	Population, *v*	Estimate, $\hat{v}$	Mean‐adjustment for weighting	
Mean	μ or μ_1_ − μ_2_	$\overset{¯}{Y}$ or ${\overset{¯}{Y}}_{1}-{\overset{¯}{Y}}_{2}$	$\frac{\sigma^{2}}{n}$	$\frac{s^{2}}{n}$	$\frac{\sum_{j=1}^{k}s_{j}^{2}/k}{n}$	*n* − 1
ln*R*	ln (μ_1_/μ_2_)	$\ln({\overset{¯}{Y}}_{1}/{\overset{¯}{Y}}_{2})$	$\frac{\sigma_{1}^{2}}{n_{1}\cdot \mu_{1}^{2}}+\frac{\sigma_{2}^{2}}{n_{2}\cdot \mu_{2}^{2}}$	$\frac{s_{1}^{2}}{n_{1}\cdot {\overset{¯}{Y}}_{1}^{2}}+\frac{s_{2}^{2}}{n_{2}\cdot {\overset{¯}{Y}}_{2}^{2}}$	$\frac{\sum_{j=1}^{k}{\left(s_{1}^{2}/{\overset{¯}{Y}}_{1}^{2}\right)}_{j}/k}{n_{1}}+\frac{\sum_{j=1}^{k}{\left(s_{2}^{2}/{\overset{¯}{Y}}_{2}^{2}\right)}_{j}/k}{n_{2}}$	*n*_1_ + *n*_2_ − 2
SMD	$\frac{\mu_{1}\;-\;\mu_{2}}{\sigma }$	*J* · *d*	$J^{2}\cdot \frac{q\cdot (1\;+\;\tilde{n}\cdot \delta^{2})}{\left(q\;-\;2\right)\cdot \tilde{n}}-\delta^{2}$	$J^{2}\cdot \left(\frac{1}{\tilde{n}}+\frac{d^{2}}{2\cdot (n_{1}+n_{2})}\right)$	$J^{2}\cdot \left(\frac{1}{\tilde{n}}+\frac{\sum_{j=1}^{k}d_{j}^{2}/k}{2(n_{1}\;+\;n_{2})}\right)$	*n*_1_ + *n*_2_ − 2

The study weights that minimize the variance of the meta‐estimate of effect size are given by 1/(*v*_*i*_ + τ^2^) for each study *i* (Hedges, 1982). These are usually estimated from the data by:

(1) $$W_{i}=\frac{1}{{\hat{v}}_{i}+T^{2}},$$

using the ${\hat{v}}_{i}$ defined in Table 2, and setting *T* to zero in the case of a fixed‐effects meta‐analysis. The weighted estimate of the meta‐effect δ from *k* studies is then:

(2) $$\text{meta‐}\hat{\delta }=\left(\sum_{i=1}^{k}W_{i}\cdot {\hat{\delta }}_{i}\right)/\sum_{i=1}^{k}W_{i}.$$

The estimate of variance in the meta‐effect is:

(3) $$\text{meta‐}\hat{v}=1/\sum_{i=1}^{k}W_{i}.$$

We wish to develop a weighting on precision that circumvents the issue described in the previous section, of asymmetric accumulation of sampling errors in the estimation of variances. We achieve this by making an adjustment to the calculation of the ${\hat{v}}_{i}$ used for weighting. The component of error variance that is itself prone to sampling error is replaced by the cross‐study mean of this component. The swap is made on the assumption that this variance component is not parametrically related to study‐specific replication and is sampled at random from the same population variance for all studies, and that the meta‐analysis includes sufficient studies for averaging. We evaluate these assumptions with simulations. Table 2 shows the study‐level estimate of error variance, and its mean‐adjustment for the one‐sample mean, and two‐sample ln*R* and SMD. For each study *i*, although the mean term (the summation divided by *k*) is a cross‐study constant, mean‐adjusted error variances remain study‐specific if studies differ in their within‐study replication. We use the inverse of this mean‐adjusted‐variance as the ‘adjusted’ weighting, and compare its performance to that of the ‘conventional’ inverse‐variance weighting. For further comparison, ‘*n*‐weighting’ replaces the mean‐adjustment term with unity to obtain what is sometimes referred to as a ‘nonparametric variance’ (e.g. Mayerhofer, Kernaghan, & Harper, 2013).

### 3.2 Meta‐analysis simulations

Datasets of study‐level means and variances were computer‐generated for simulations to evaluate bias in one‐ and two‐sample estimators, using comparable ranges of within‐study replication. Each of the *n*_*i*_ observations in a study sample was drawn from a normal distribution with *SD* σ, and mean μ (fixed effects) or mean μ_*i*_ (random effects, itself drawn from a normal distribution of $\delta_{i}$ with mean δ and *SD* τ). Study‐level parameters δ and *v*_*i*_, and their estimates ${\hat{\delta }}_{i}$ and ${\hat{v}}_{i}$ were calculated according to the Table‐2 formulae. For ln*R*, population means were set with the same sign, and far enough above zero to avoid a known small‐sample bias with near‐zero means (Hedges et al., 1999; confirmed with simulations using the small‐sample correction by Lajeunesse, 2015). Study weightings were calculated by Equation (1). Random‐effects meta‐analysis measured the component of error variance due to variability between studies as the parameter value τ^2^, in order to avoid confounding error by an estimate *T* ^2^ obtained from any of the many data‐based methods (reviewed in Veroniki et al., 2016). We separately evaluated the influence of weighting type on the two most common *T* ^2^ estimators, calculated by the method of moments, and by maximum likelihood (DerSimonian‐Laird and REML, respectively, in R package ‘metafor’: Viechtbauer, 2010).

Meta‐analysis of each simulated dataset was the same for all estimators and types of weighting. The meta‐effect, $\text{meta‐}\hat{\delta }$, was estimated by Equation (2). The meta‐effect variance, $\text{meta‐}\hat{v}$, was estimated by Equation (3). The accuracy of the meta‐effect was measured as $|\text{meta‐}\hat{\delta }-\delta |$. The significance of the meta‐effect was estimated from Student's *t* = $\text{meta ‐}\hat{\delta }/\sqrt{\text{meta‐}\hat{v}}$, and evaluated against the parameter‐based Student's *t* = $\delta /\sqrt{\text{meta‐}v}$ (both having *k *− 1 d.f.). Each output value was reported as the median of 10,000 trial runs. Data S1 lists the r script for the simulations.

### 3.3 Empirical examples

We ran simulations on input parameter values derived from three empirical studies. As above, each simulation was repeated 10,000 times to evaluate meta‐estimates from conventional and adjusted weightings. In addition, meta‐analyses were run on the three sets of published study‐specific ${\left\{{\overset{¯}{Y}}_{1},\;{\overset{¯}{Y}}_{2}\right\}}_{i}$ and ${\left\{s_{1},\;s_{2}\right\}}_{i}$, to compare results by the two types of weighting. Data S2 lists the r script for meta‐analysis.

A meta‐analysis of 165 studies was simulated with the same replication as a fixed‐effects meta‐analysis by Ma and Chen (2016), which estimated effects of species diversity on fine root biomass as a log response ratio between mixed and monoculture forests. Studies had widely ranging sample sizes {*n*_1_, *n*_2_}_*i*_ from {56, 128} downwards, including 57 × {4, 4}, 45 × {3, 3}, 13 × {2, 2}, and 5 × {1, 1}. Cross‐study means of within‐study ${\overset{¯}{Y}}_{1},\;\;{\overset{¯}{Y}}_{2}$ and *s*_1_, *s*_2_ were used to define the otherwise unknown true parameter values for input to the simulation: μ_1_ = 368.4, μ_2_ = 300.8, σ_1_ = 129.8, σ_2_ = 134.6.

A meta‐analysis of 23 studies was simulated with the same sample sizes as a random‐effects meta‐analysis by Gibson et al. (2011) estimating bird abundance and richness in abandoned agriculture as a standardized mean difference from primary forest: {*n*_1_, *n*_2_}_*i*_ = 4 × {12, 12}, 3 × {6, 3}, 16 × {2, 2}. The empirical data had $\text{meta‐}\hat{\delta }$ = −2.27 and −15.09 for Hedges’ *g* and ${\overset{¯}{Y}}_{1}-{\overset{¯}{Y}}_{2}$, respectively, and *T* ^2^ = 2.39 given by the method of moments. These estimates were used to define input parameters δ = −2.27, and σ = 6.66 and τ = 1.55 in simulation runs as described above. A set of 1,000 runs was first used to obtain REML estimates of τ for each type of weighting; the simulation was then run 10,000 times to obtain meta‐estimates from each type of weighting using their associated REML estimates of τ.

The simulation was repeated for a meta‐analysis of 65 studies with the same sample sizes as those of Gibson et al. (2011) for meta‐estimation of bird abundance and richness in active agriculture as a standardized mean difference from primary forest: {*n*_1_, *n*_2_}_*i*_ = 20 × {50, 25}, 10 × {50, 4}, 7 × {12, 12}, 15 × {6, 6}, 13 × {4, 4}. The empirical data had $\text{meta‐}\hat{\delta }$ = −1.42 and −4.78 for Hedges’ *g* and ${\overset{¯}{Y}}_{1}-{\overset{¯}{Y}}_{2}$, respectively, and *T* ^2^ = 6.53. These estimates provided simulation inputs δ = −1.42, σ = 3.36 and τ = 2.56. Meta‐estimates for each type of weighting were again obtained using their associated REML estimates of τ.

## 4 RESULTS

### 4.1 Simulations of equally replicated studies

For meta‐analysis of a one‐sample mean, conventional weighting of 20 studies all with the same *n*_*i*_ = 10 observations strongly overvalued the precision of the meta‐effect. Figure 3(a) illustrates the source of bias, in *n*‐dependent sampling error causing ${\hat{v}}_{i}$ to vary around *v*. The upper‐right plot shows the inverse of the harmonic mean (in blue) of the estimated variances exceeding the inverse of the parametric variance. The conventional weighting consequently undervalued the meta‐variance by 21%. Table 3(a) enumerates the biases, showing that although this weighting gave an unbiased meta‐effect, it inflated the significance of the effect. The adjusted weighting resolved the undervaluation of the meta‐variance, as illustrated in the lower‐right graph of Figure 3(a), and hence also the inflated significance as enumerated in Table 3(a). The tabulation further shows this weighting achieving an improvement in the accuracy of the meta‐effect. The same improvement in accuracy was achieved by *n*‐weighting, which, however, could not estimate the meta‐variance or the significance of the meta‐effect. These differences between the weightings changed negligibly with the magnitudes of μ or σ^2^, or their study‐level specificities.

**Figure 3:**
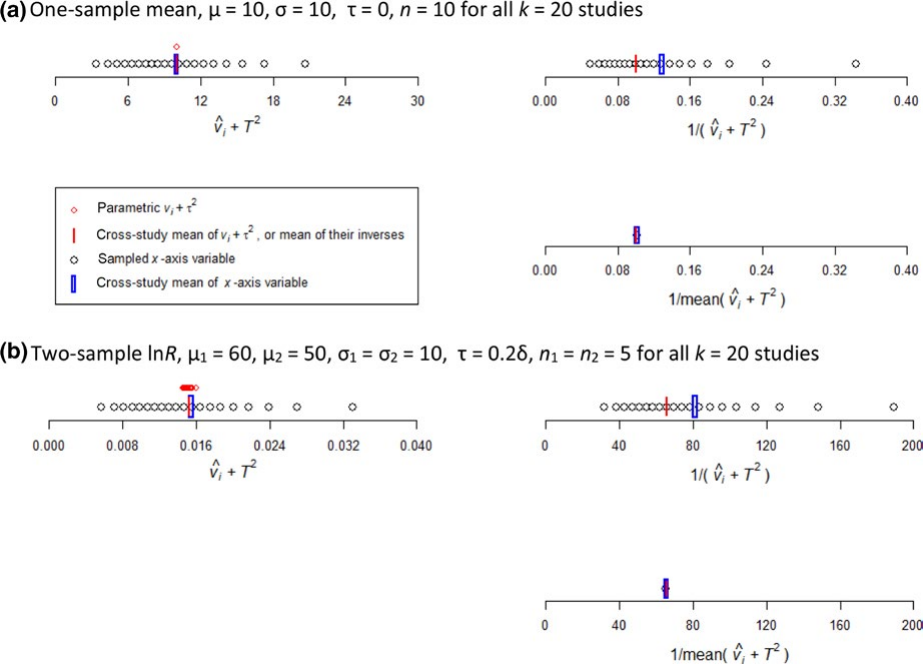
Source of biases in precision‐weighted meta‐analysis of equally replicated studies. (a) For a one‐sample mean with fixed effects, left‐hand graph shows the mean of the study‐level ${\hat{v}}_{i}$ coinciding with the parametric *v* (blue and red lines, respectively), despite some right skew caused by ${\hat{v}}_{i}$ being constrained to positive values. Upper right‐hand graph shows the conventional weighting averaged across studies (blue line) substantially exceeding the inverse of the parametric *v* (red line). Lower right‐hand graph shows the weighting by inverse adjusted‐variance coinciding with 1/*v*. (b) A two‐sample ln*R* with random effects likewise shows bias induced by conventional weighting on precision, and diminished by adjusted weighting. All points and means are averaged over 10,000 trials

**Table 3:** Simulations of precision‐weighted meta‐analyses on *k* = 20 studies. Parts (a)**–**(c) enumerate outputs from Figure 3a, b and Figure S2c. Results with the weighting by inverse‐variance in column 1, and by inverse adjusted‐variance in column 2. *identifies best estimate, *^=^identifies equal‐best estimate

Estimator and test	Study‐level ${\hat{v}}_{i}$	Mean‐adjusted ${\hat{v}}_{i}$
(a) One‐sample mean, μ = 10, σ = 10, τ = 0, *n* = 10 for all studies		
Ratio estimated: parametric meta‐effect	1.00*^=^	1.00*^=^
Accuracy: $|\text{meta‐}\hat{\delta }-\delta |$	0.551	0.488*
Ratio estimated: parametric meta‐variance	0.79	1.00*
Ratio estimated: parametric Student's *t*	1.12	1.00*
(b) Two‐sample ln*R*, μ_1_ = 60, μ_2_ = 50, σ_1_ = σ_2_ = 10, τ = 0.2δ, *n*_1_ = *n*_2_ = 5 for all studies		
Ratio estimated: parametric meta‐effect	0.99*^=^	1.01*^=^
Accuracy: $|\text{meta‐}\hat{\delta }-\delta |$	0.020	0.019*
Ratio estimated: parametric meta‐variance	0.82	1.02*
Ratio estimated: parametric Student's *t*	1.09	1.00*
(c) Two‐sample SMD, μ_1_ = 60, μ_2_ = 50, σ_pooled_ = 10, τ = 0.2δ, *n*_1_ = n_2_ = random 3 to 10		
Ratio estimated: parametric meta‐effect	0.91	1.00*
Accuracy: $|\text{meta‐}\hat{\delta }-\delta |$	0.111	0.101*
Ratio estimated: parametric meta‐variance	0.97*^=^	0.97*^=^
Ratio estimated: parametric Student's *t*	0.92	1.01*

Likewise for the two‐sample ln*R*, conventional weighting undervalued the meta‐variance due to *n*‐dependent sampling error in variance estimation. Adjusted weighting largely corrected the bias, as illustrated in Figure 3(b) and enumerated in Table 3(b). These differences were little influenced by the presence of moderate between‐study variability in population means, shown here at τ* = *0.2δ; at a larger τ* = *δ, however, the much larger between‐ than within‐study variance rendered all weightings similar and suppressed differences between the weighting types.

Conventional weighting undervalued the meta‐variance according to an inverse function of *n* for the one‐sample mean and two‐sample ln*R*, as illustrated in Figure 4 (red circles and dots, right‐hand graphs). For Hedges’ *g*, conventional weighting principally undervalued the meta‐effect by an inverse function of *n* (Figure 4 red crosses, left‐hand graphs). The bias is a predictable consequence, recognized by Hedges (1982), of the positive contribution that the estimated magnitude of effect $|{\hat{\delta }}_{i}|$ makes to the estimated error variance ${\hat{v}}_{i}$ (Table 2), resulting in deviations below |δ| achieving higher weighting. The increase in bias with 1/*n* reflects the *n*‐dependent sampling error in estimating the two sample means and the standard deviations of observations around them. Adjusted weighting effectively corrected this bias in the meta‐effect, as well as eliminating or reducing biases in the meta‐variances of the one‐sample mean and two‐sample ln*R* (Figure 4, blue symbols closer to zero than red symbols).

**Figure 4:**
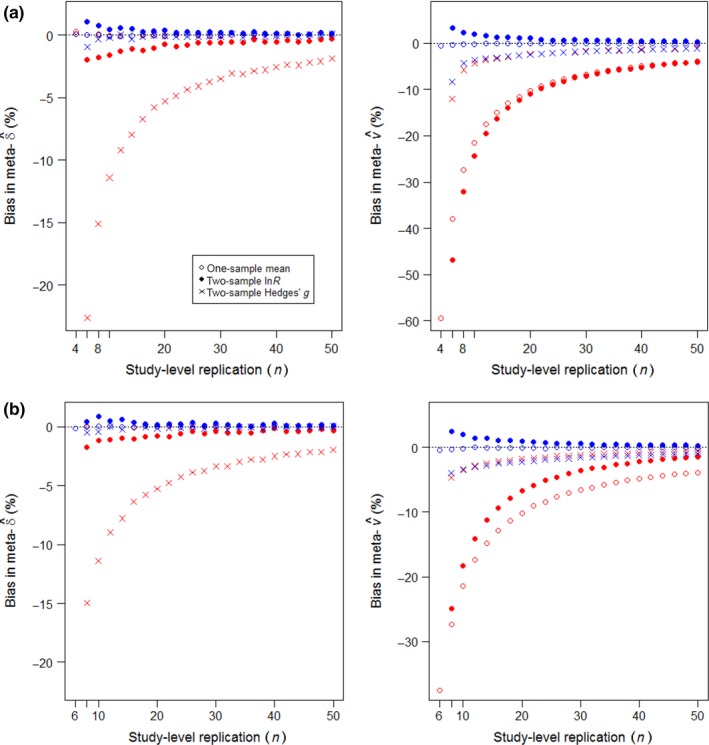
Replication‐dependent bias in meta‐estimations from precision‐weighting on *k* = 40 studies. Red symbols show estimation by conventional weighting; blue symbols by adjusted weighting, each the median of 10,000 runs. (a) Fixed‐effects meta‐analysis (τ = 0); (b) random‐effects meta‐analysis (τ = 0.2δ). Bias = 100 × (estimate/parameter − 1), with negative values signifying undervaluation. Input parameters: μ = 10 for one‐sample studies, or μ_1_ = 60, μ_2_ = 50 for two‐sample studies; σ = 10; sample sizes *n* for one‐sample studies, or *n*_1_ = *n*_2_ = *n*/2 for two‐sample studies, equal across all *k* studies

The Figure 4 simulations all ran on *k* = 40 studies; when repeated on *k* = 4 studies, they obtained a similar quality of response (Figure S1). For the random‐effects meta‐analysis of ln*R*, the small number of studies slightly reduced the magnitude of undervaluation of the meta‐variance caused by conventional weighting, and improved the correction by adjusted weighting. For other estimators, it slightly degraded the correction by adjusted weighting at very low replication, which nevertheless still improved on conventional weighting.

In further trials with Hedges’ *g*, larger magnitudes of δ reduced the undervaluation of the meta‐variance for both types of weighting, and switched to causing overvaluation when |δ| exceeded *c*. 1.25, regardless of replication and always with less bias for the adjusted than conventional weighting. Larger magnitudes of δ had no impact on the undervaluing of the meta‐effect by conventional weighting or its correction by weighting with inverse adjusted‐variance. For the one‐sample mean and ln*R*, larger magnitudes of δ made no discernible impacts on undervaluation of meta‐variances or meta‐effects.

### 4.2 Simulations of variably replicated studies

For meta‐analyses that encompassed variably replicated studies, all three estimators gave equivalent results to those for equal replication. Figure 5 shows the simulations producing the same quality and similar magnitudes of bias due to conventional weighting, and benefits in adjusted weighting (cf. Figure 4). Full outputs for the Figure 3 examples when they had variably replicated studies are provided in Figure S2 and Table S1. Conventional weighting of Hedges’ *g* again showed bias principally in the meta‐effect (Figure 5), which then had consequences for its significance, as enumerated for a random‐effects example in Table 3(c) (also illustrated in Figure S2c). For all estimators, *n*‐weighting matched adjusted weighting for resolving bias in the meta‐effect and improving accuracy, but could not estimate a meaningful meta‐variance necessary for testing significance.

**Figure 5:**
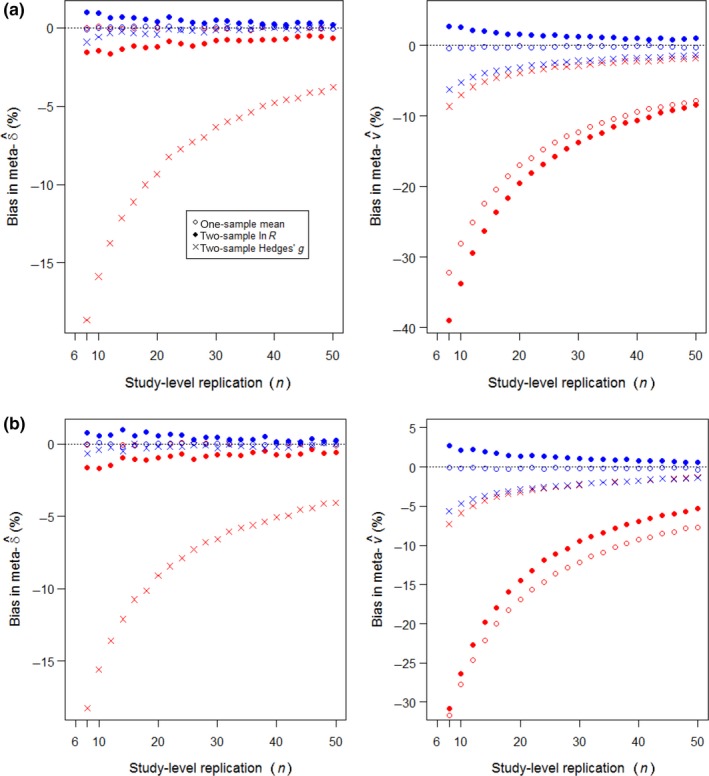
Replication‐dependent bias in estimations from precision‐weighted meta‐analysis. Symbols and parameters as for Figure 4, except the *k* = 40 studies have replication varying randomly between 6 and *n* observations. (a) Fixed effects; (b) random effects

For the ln*R* estimator, the adjusted weighting was also effective in reducing known issues of small‐sample bias, when one of the two μ takes a value close to zero (Lajeunesse, 2015). With μ_1_ = 0.5, μ_2_ = 2 and σ = 0.2, τ* *= 0, conventional weighting on 20 studies using *n*_1_ = *n*_2_ = 2 vastly overvalued the significance of the meta‐effect (*t*_19_ = 57.69, compared to parametric *t*_19_ = 21.27), due to undervaluation of the meta‐variance by 87% combined with slight undervaluation of the meta‐effect (by 3%). The adjusted weighting completely eliminated this large bias in the meta‐variance (estimate:parameter = 1.00), reducing the significance of the meta‐effect to a slight undervaluation (*t*_19_ = 19.60).

DerSimonian‐Laird and REML estimates of between‐sample variance produced similar biases in τ according to the type of weighting (Table S2). For one‐sample mean and two‐sample ln*R* estimators, both methods over‐ and undervalued magnitudes of τ by conventional and adjusted weightings, respectively; the adjusted weighting consistently achieved a value closer to the true τ, and substantially closer at low study‐level replication. These directions of bias were switched for two‐sample SMD at low study‐level replication, with the conventional weighting achieving a value closer to the true τ.

### 4.3 Empirically derived simulations and meta‐analyses

The simulation of fine root responses to species diversity was typical of many ecological meta‐analyses in encompassing numerous little‐replicated studies. Its sample sizes predicted an undervaluation of the ln*R* meta‐effect by 5% with conventional weighting. This weighting also undervalued the meta‐variance by 23%, causing an overvaluation of significance. The adjusted weighting reduced the magnitude of bias in the meta‐effect to a 2% overvaluation. It improved the accuracy, from $|\text{meta‐}\hat{\delta }-\delta |$ = 0.019 to 0.011. It eliminated bias in the meta‐variance (estimate:parameter = 1.00), although a mean overvaluation (caused by the high values of σ) slightly undervalued significance, resulting nevertheless in an overall improvement in the estimate (from *t* = 14.30 originally to 12.54, compared to parametric *t* = 13.32). When the adjusted weighting was applied to fixed‐effects meta‐analysis of the study‐specific ${\left\{{\overset{¯}{Y}}_{1},\;{\overset{¯}{Y}}_{2}\right\}}_{i}$ and ${\left\{s_{1},\;s_{2}\right\}}_{i}$ published in Ma and Chen (2016), it increased the magnitude of the meta‐effect by 42% over that given by conventional weighting. It increased the meta‐variance by 356%, resulting in a reduction in significance from *t*_164_ = 23.52 to *t*_169_ = 15.67. Note that the adjusted weighting used an additional five studies, which were not available to the conventional weighting because they had no estimates of within‐study *s*. For those additional studies, the components of error variance not due to replication were estimated from their means across all of the subset of studies that did provide values of *s*.

The simulation of bird responses to abandoned agriculture typified many ecological meta‐analyses in having large between‐study variability. Its sample sizes predicted an 11% undervaluation of the magnitude of the Hedges’ *g* meta‐effect by conventional weighting. This was resolved by the adjusted weighting which had an undervaluation of just 0.4%. The adjusted weighting also improved accuracy by 72% ($|\text{meta‐}\hat{\delta }-\delta |$ = 0.147 compared to 0.254 for conventional weighting). These improvements obtained from adjusted and conventional weightings that substantially over‐ and underestimating τ, at 44.7 and 0.30 respectively. Both weightings overvalued the meta‐variance (by 194% and 136%, due to the high magnitude of δ ≫ 1.25, and the overestimation of τ for adjusted weighting), causing both to undervalue the significance of the effect (*t* = 12.33 and 13.44, for parametric *t* = 17.62). The best accuracy and significance was achieved by meta‐estimation with adjusted weighting using conventionally weighted estimation of τ ($|\text{meta‐}\hat{\delta }-\delta |$ = 0.127, and thus twice the accuracy of conventional weighting, and *t* = 14.65), which aligned closely with adjusted weighting using the true τ = 1.55 ($|\text{meta ‐}\hat{\delta }-\delta |$ = 0.129, *t* = 14.62). When applied to random‐effects meta‐analysis of the study‐specific data published in Gibson et al. (2011), the adjusted weighting increased the magnitude of the meta‐effect by 14% over that given by conventional weighting, and increased the meta‐variance by 9%, resulting in an increase in significance from *t*_22_ = 6.34 to *t*_22_ = 6.92.

Differences due to weighting were less apparent for the simulation of bird responses to active agriculture, with nearly three times the number of studies, better replication, smaller δ and larger τ. Conventional weighting obtained a 3% undervaluation of the magnitude of meta‐effect. This was again resolved by the adjusted weighting which had <0.1% undervaluation. The adjusted weighting also predicted higher accuracy ($|\text{meta ‐}\hat{\delta }-\delta |$ = 0.036 compared to 0.050 by conventional weighting). These improvements obtained from adjusted and conventional weightings that again over‐ and underestimating τ, at 10.4 and 0.30. Both weightings slightly overvalued the meta‐variance (by 1% for both weighting types), causing both to undervalue the significance of the effect (*t* = 27.37 and 27.98, for parametric *t* = 28.58). The best accuracy and significance was again achieved by meta‐estimation with adjusted weighting using conventionally weighted estimation of τ ($|\text{meta‐}\hat{\delta }-\delta |$ = 0.035, *t* = 28.81), which aligned closely with adjusted weighting using the true τ = 2.56 ($|\text{meta ‐}\hat{\delta }-\delta |$ = 0.035, *t* = 28.70). When applied to random‐effects meta‐analysis of the study‐specific data published in Gibson et al. (2011), the adjusted weighting increased the magnitude of the meta‐effect by 20% over that given by conventional weighting, and increased the meta‐variance by 1%, resulting in an increase in significance from *t*_64_ = 4.47 to *t*_64_ = 5.35.

## 5 DISCUSSION

Modern statistics of model comparison attach greater importance to magnitudes of effects than to significance (Hector, 2015). Of the 25 published meta‐analyses enumerated in Figure 1, the abstracts varied in their emphases on magnitude or significance. Thirteen presented abstracts that focused principally on effect sizes, and therefore concerned the accuracy of meta‐estimation; ten focused principally on the significance of effects, and therefore concerned the precision of meta‐estimation; and two concerned both. Fourteen of the 25 obtained meta‐estimates from inverse‐variance weighting, including seven of the fifteen that were not focused only on significance. Here, we have shown how this weighting undervalues the meta‐variance particularly for a one‐sample mean and two‐sample ln*R*, and the meta‐effect for the two‐sample Hedges’ *g*, and thereby systematically influences the accuracy and the significance of meta‐estimations. Although studies may use other weights than the error variance for a Gaussian distribution (Gurevitch et al., 2001), significance inflation (for one‐sample mean and ln*R*) or deflation (Hedges’ *g*) can result from weighting on any unbiased estimator of study‐level error variances. It arises wherever the estimate of meta‐variance derives from a harmonic mean of study‐specific estimates of error variance that are prone to sampling error. Moreover, it becomes substantial in meta‐analyses of little‐replicated studies if the variance in study‐level observations depends on study‐level replication, which will almost certainly be the case.

We have demonstrated that weighting on study precision by inverse adjusted‐variance will eliminate or substantially reduce biases due to low study‐level replication. The adjustment, by cross‐study averaging of the component of error variance that is itself prone to sampling error, universally improved accuracy in estimation of both the meta‐effect and its significance. It was effective in dealing with known issues of small‐sample bias in Hedges’ *g* (Hedges, 1982) and in ln*R* (Hedges et al., 1999; Lajeunesse, 2015). It had the great advantage over *n*‐weighting of producing valid estimates of meta‐variance and significance of the meta‐effect. The adjusted weighting would be relevant also to heterogeneity statistics *Q*_*T*_ and *I* ^2^, which derive from weighted effect‐size estimation. It is not relevant to meta‐analyses on a correlation effect, which uses 1/(*n *− 3) for the variance of Fisher's *z*‐transformation of *r*, and therefore has no variance component prone to sampling error.

For random‐effects meta‐analysis, we found that between‐study variation generally had little influence on the magnitude of replication‐dependent bias and the effectiveness of its correction by adjusted weighting. Variability in *T* ^2^ estimating τ^2^ nevertheless increases with the inverse of *k*, just as *s*_*i*_^2^ estimating σ_*i*_^2^ increases with the inverse of *n*_*i*_ (Figure 2a), and bias in *T* ^2^ increases with the inclusion of more little‐replicated studies (Table S2). For the one‐sample mean and two‐sample ln*R*, adjusted weighting gave the least bias in *T* ^2^. For meta‐estimation of little‐replicated studies with Hedges’ *g*, we recommend using adjusted weighting for the meta‐effect and meta‐variance following conventionally weighted DerSimonian‐Laird or REML estimation of τ^2^. This avoids the overvaluing of τ^2^ by adjusted weighting specifically for applications of SMD to little‐replicated studies, although an adjusted‐weighting estimate of τ^2^ would barely degrade the accuracy of the meta‐effect.

In conclusion, we see no reason not to adopt adjusted weighting for all meta‐analyses that are concerned with accurately and precisely estimating a meta‐effect. It addresses a bias that applies even when studies are all well‐replicated, albeit with much less influence than at small sample sizes. Moreover, it can expand the scope of meta‐analyses to include some studies that lack variance estimates, a common occurrence in primary studies (Gerstner et al., 2017), on the assumption that all observations are sampled from the same global σ. The r script in Data S2 will calculate the mean‐adjusted study‐level error variance ${\hat{v}}_{i}$ for weighted meta‐estimation of a one‐sample mean, or the two‐sample ln*R* or Hedges’ *g*, given study‐level information on the sample size(s), effect size and the variance(s) in observations.

## ACKNOWLEDGEMENTS

We thank Julia Koricheva for allowing us to use data from Koricheva and Gurevitch (2014) for Figure 1. We thank Robert B. O'Hara and Dankmar Boehning for clarifying key issues in earlier versions, and two anonymous reviewers for constructive comments. The Biotechnology and Biological Sciences Research Council provided funding to R.S. (grant no. BB/H531935/1).

## AUTHORS’ CONTRIBUTIONS

C.P.D. conceived the idea, designed the methodology, analysed and interpreted the data, and led the writing of the manuscript. R.S. contributed to interpretation and writing of main text.

## DATA ACCESSIBILITY

R scripts for simulations in Data S1, and for meta‐analysis with adjusted weighting in Data S2, are deposited in the Dryad Digital Repository http://datadryad.org/resource/doi:10.5061/dryad.5f4g6 (Doncaster & Spake, 2017).

## Supporting information


**Figure S1**

**Figure S2**

**Table S1**

**Table S2**

**Data S1**

**Data S2**
