# Supplementary material for: Correction for bias in meta‐analysis of little‐replicated studies
Source: Methods Ecol Evol. 2017 Nov 21;9(3):634–44. doi: 10.1111/2041-210X.12927 (PMC5993351; doi:10.1111/2041-210X.12927)
Supplement: Supplementary file 1 — Figure S1 [file MEE3-9-634-s001.pdf]

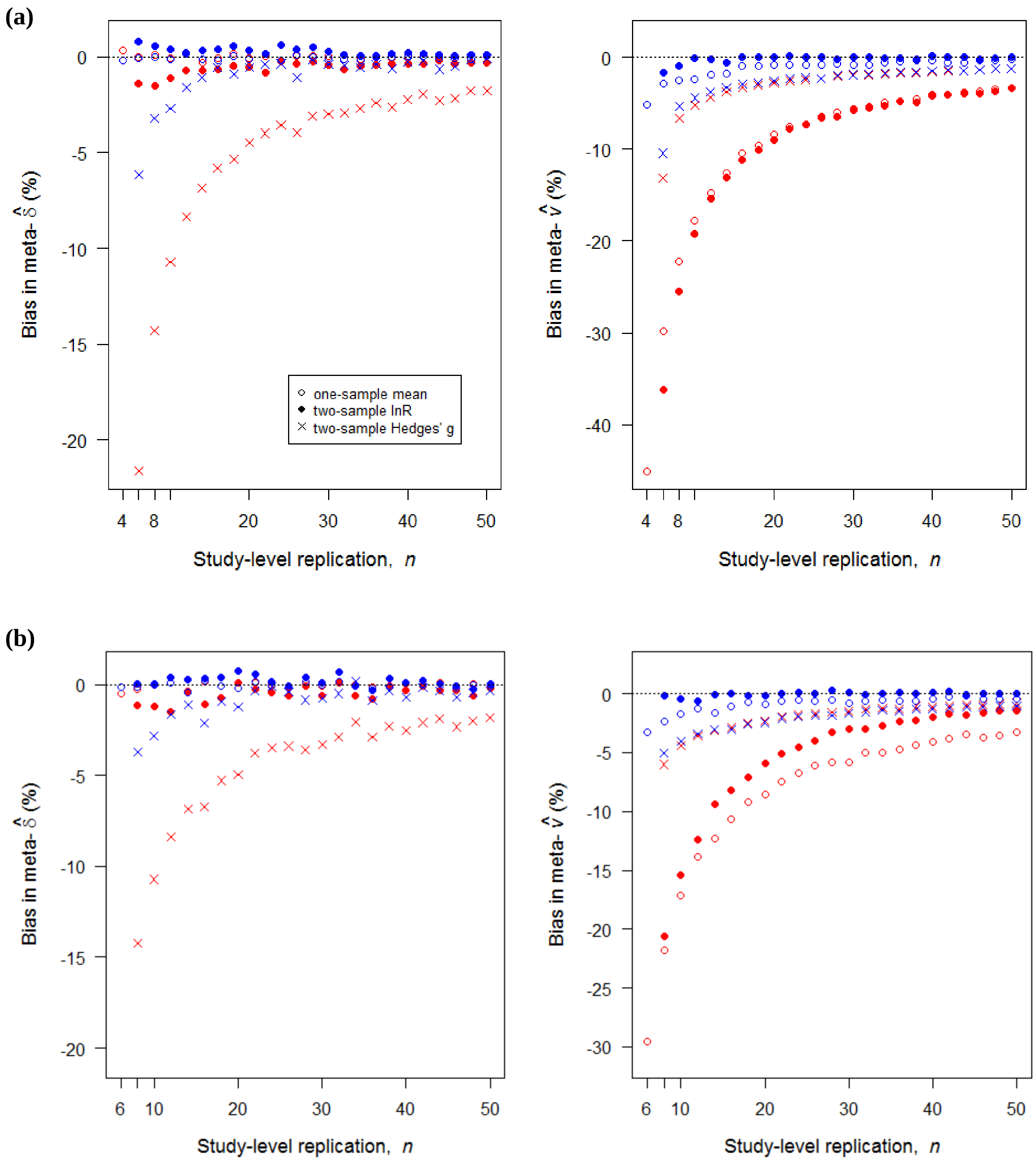

Fig. S1. Replication-dependent bias in estimations from precision-weighted meta-analysis. Symbols and parameters as for main-text Fig. 4, except k = 4 studies. **(a)** Fixed effects; **(b)** random effects.
