# Supplementary material for: Correction for bias in meta‐analysis of little‐replicated studies
Source: Methods Ecol Evol. 2017 Nov 21;9(3):634–44. doi: 10.1111/2041-210X.12927 (PMC5993351; doi:10.1111/2041-210X.12927)
Supplement: Supplementary file 2 — Figure S2 [file MEE3-9-634-s002.pdf]

(a) One-sample mean, μ = 10, σ = 10, τ = 0.2δ, n = random between 3 and 20

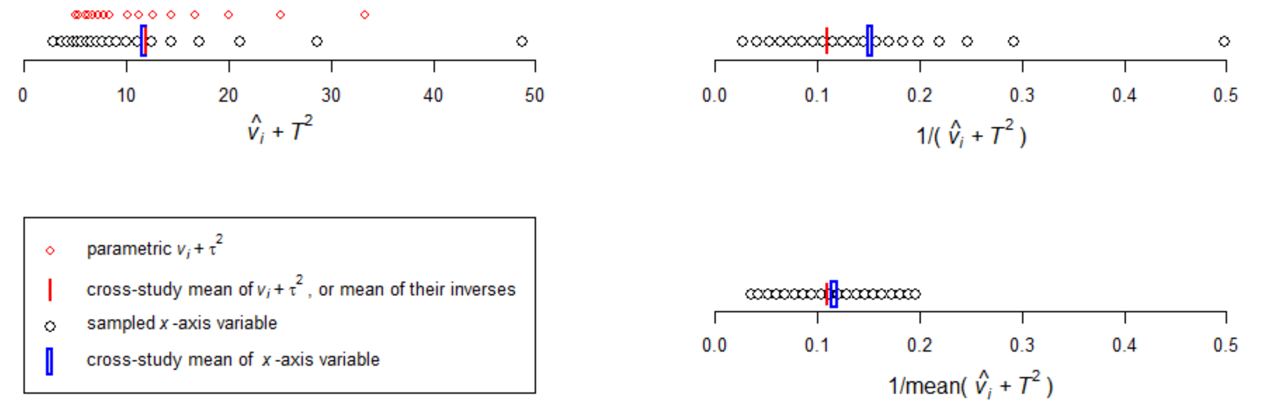

(b) Two-sample ln$R$, μ_1 = 60, μ_2 = 50, $\sigma_1 = \sigma_2$ = 10, τ = 0.2δ, $n_1 = n_2$ = random 3 to 10

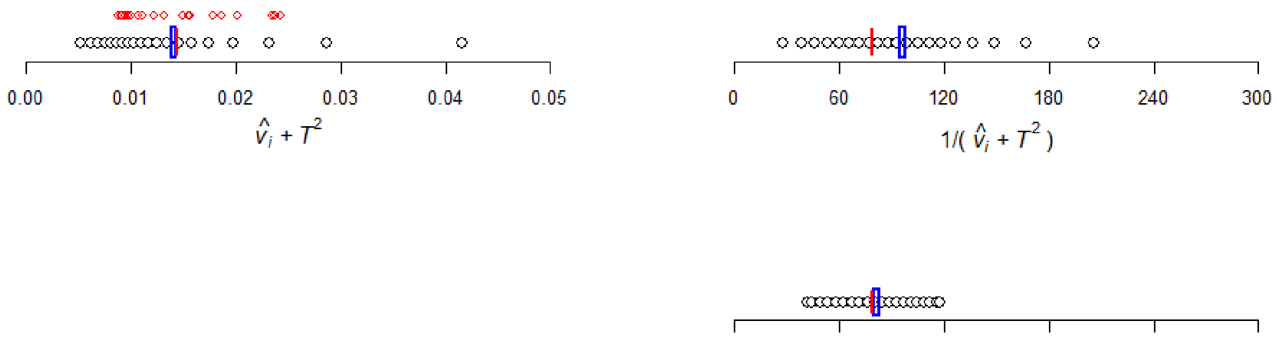

(c) Two-sample Hedges' g, μ_1 = 60, μ_2 = 50, σ_{pooled} = 10, τ = 0.2δ, $n_1 = n_2$ = random 3 to 10

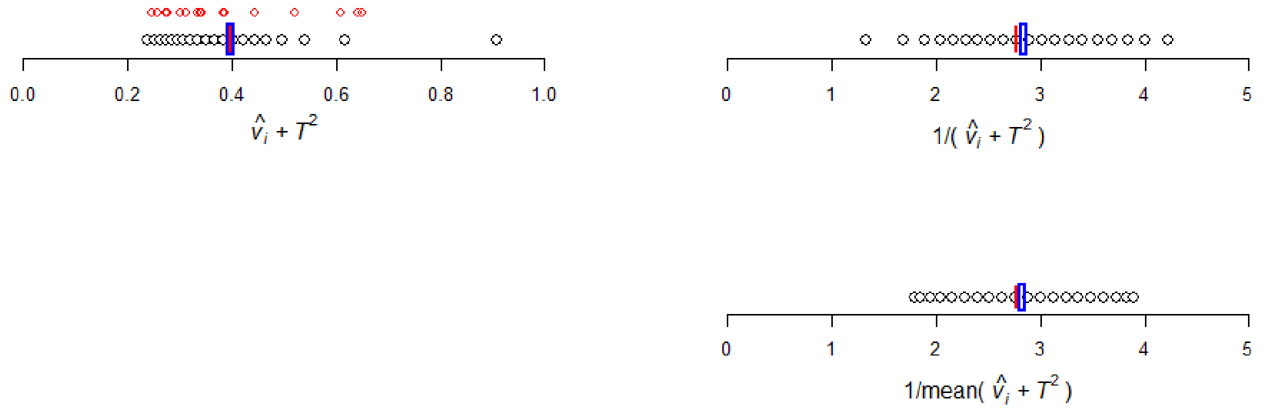

Fig. S2. Source of biases in precision-weighted meta-analysis of variably-replicated studies. For each of the three estimators on k = 20 studies, graphs in **(a)-(c)** show error variances (left-hand), and weighting on precision by inverse-variance (upper right-hand) or inverse-mean-variance (lower right-hand). All points and means are averaged over 10,000 trials. Note that weightings by inverse-adjusted-variance are study-specific, reflecting their study-specific n_i (in contrast to main-text Fig. 3).
