# Supplementary material for: Correction for bias in meta‐analysis of little‐replicated studies
Source: Methods Ecol Evol. 2017 Nov 21;9(3):634–44. doi: 10.1111/2041-210X.12927 (PMC5993351; doi:10.1111/2041-210X.12927)
Supplement: Supplementary file 3 — Table S1 [file MEE3-9-634-s003.pdf]

Table S1. Simulations of precision-weighted meta-analysis. Results with weighting by inverse-variance in column 1, and by inverse-adjusted-variance in column 2. * identifies best estimate.

Estimator and test	Study-level $\hat{v}_i$	Mean-adjusted $\hat{v}_i$
Fig. S2(a) one-sample mean as for Fig. 3(a), except τ = 0.2, n = random 3 to 20		
Ratio estimated : parametric meta-effect	1.00*=	1.00*=
Accuracy: $\left\|\text{meta-}\hat{\delta} - \delta\right\|$	0.558	0.453*
Ratio estimated : parametric meta-variance	0.80	1.00*
Ratio estimated : parametric Student's *t*	1.12	1.00*
*Fig. S2(b) two-sample ln*R *as for Fig. 3(b), except $n_1 = n_2$ = random 3 to 10*		
Ratio estimated : parametric meta-effect	0.99	1.00*
Accuracy: $\left\|\text{meta-}\hat{\delta} - \delta\right\|$	0.019	0.017*
Ratio estimated : parametric meta-variance	0.86	1.01*
Ratio estimated : parametric Student's *t*	1.07	1.00*
Fig. S2(b) variant with τ = 0		
Ratio estimated : parametric meta-effect	0.99	1.00*
Accuracy: $\left\|\text{meta-}\hat{\delta} - \delta\right\|$	0.018	0.015*
Ratio estimated : parametric meta-variance	0.81	1.01*
Ratio estimated : parametric Student's *t*	1.09	0.99*
