# Supplementary material for: Correction for bias in meta‐analysis of little‐replicated studies
Source: Methods Ecol Evol. 2017 Nov 21;9(3):634–44. doi: 10.1111/2041-210X.12927 (PMC5993351; doi:10.1111/2041-210X.12927)
Supplement: Supplementary file 4 — Table S2 [file MEE3-9-634-s004.pdf]

Table S2. Evaluation of DerSimonian-Laird and REML estimates of τ at values equal to 0.4δ, 0.2δ and 0, using conventional and adjusted weighting, from medians of 1000 simulation runs.

* identifies best estimate.

		DerSimonian-Laird		REML	
n_i	τ	Conventional	Adjusted	Conventional	Adjusted
One-sample mean, with μ *= 10,* σ *= 10,* k *= 50*					
100	0.4	0.410	0.392*$^{=}$	0.429	0.392*$^{=}$
100	0.2	0.209	0.150*$^{=}$	0.243	0.150*$^{=}$
100	0.0	0.145	0.080*$^{=}$	0.178	0.080*$^{=}$
3-20	0.4	1.592	0.258	1.705	0.382*
3-20	0.2	1.629	0.182*	1.742	0.003
3-20	0.0	1.636	0.000*	1.737	0.002
*Two-sample ln*R*, with* μ_1 *= 60,* μ_2 *= 50,* σ *= 10,* k *= 50*					
50, 50	0.08	0.080*$^{=}$	0.080*$^{=}$	0.080*$^{=}$	0.080*$^{=}$
3-10, 3-10	0.08	0.090	0.077*$^{=}$	0.090	0.077*$^{=}$
3-10, 3-10	0.04	0.061	0.035*$^{=}$	0.064	0.035*$^{=}$
3-10, 3-10	0.00	0.045	0.000*	0.051	0.002
Two-sample SMD, with μ_1 *= 60,* μ_2 *= 50,* σ *= 10,* k *= 50*					
50, 50	0.2	0.196	0.201*$^{=}$	0.196	0.201*$^{=}$
50, 50	0.0	0.012	0.029	0.002*	0.029
3-10, 3-10	0.2	0.232*	0.348	0.150	0.339
3-10, 3-10	0.0	0.148	0.288	0.002*	0.270
